# The identification of eosinophilic gastroenteritis in prednisone-dependent eosinophilic bronchitis and asthma

**DOI:** 10.1186/1710-1492-7-4

**Published:** 2011-03-01

**Authors:** Parameswaran Nair, Sergei I Ochkur, Cheryl Protheroe, Elizabeth Simms, Nancy A Lee, James J Lee

**Affiliations:** 1 Firestone Institute for Respiratory Health, St. Joseph's Healthcare and Department of Medicine, McMaster University, Hamilton, Ontario, Canada; 2 Division of Pulmonary Medicine, Mayo Clinic, Scottsdale, AZ, USA

## Abstract

This case reports the unique association of eosinophilic gastrointestinal disease with eosinophilic bronchitis, asthma and chronic rhinosinusitis and some features of lymphocytic hypereosinophilic syndrome, describes a diagnostic protocol for patients with asthma and persistent eosinophilic bronchitis, and suggests that the use of a novel *EPX-mAb *provides a reliable method to identify eosinophilic inflammation.

## Introduction

Eosinophilic gastrointestinal disease (EGID) is characterized by identification of abnormal eosinophilic infiltration on morphologic evaluation of gastrointestinal tissues obtained by biopsy or resection from patients with gastrointestinal complaints [1]. EGIDs are classified according to the site involved (i.e., esophageal, gastric, small intestinal, colonic, or multiple). Esophagus is increasingly being recognized as a site of involvement with eosinophils accumulating in the mucosal, muscular, serosal, diffuse, or transmural areas [2]. The diagnosis for eosinophilic esophagitis and other EGIDs is established after ruling out other causes of an eosinophilic disease, particularly atopy, parasitic infestations, vasculitis, and hypereosinophilic syndrome (HES) [3]. We report the association of eosinophilic gastroenteritis and eosinophilic bronchitis in a young patient with prednisone-dependent asthma and some features of lymphocytic hypereosinophilic syndrome and the sensitivity of a novel monoclonal antibody directed against eosinophil peroxidase (*EPX-mAb*) [4] as an unambiguous means with which to detect both infiltrating tissue eosinophils and eosinophil degranulation in gastrointestinal tract biopsies. The patient provided written informed consent for publishing this manuscript.

## Case report

**Figure 1:**
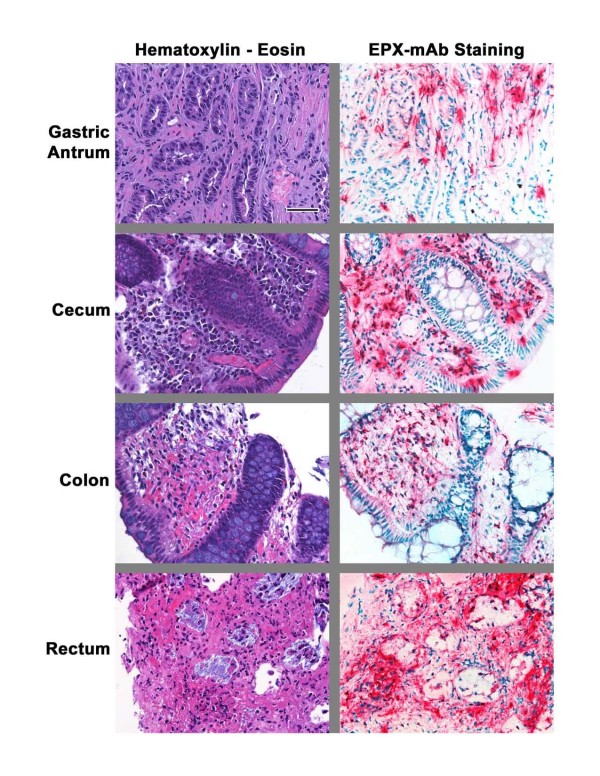
**H&E and anti-EPX staining of GI tissues**. *EPX-mAb*-based immunohistochemistry provided evidence of both tissue infiltrating eosinophils and eosinophil degranulation in GI biopsies from the patient described in this cse report. In contrast to sections stained with Hematoxylin-Eosin (left panels) which displayed only nominal evidence of eosinophil infiltration and degranulation, serial sections subjected to *EPX-mAb*-based immunohistochemistry (right panels) displayed significant evidence (magenta staining areas) of both eosinophil infiltration and degranulation (extra-cellular deposition of granules and/or free-EPX within the tissue matrix). Each photomicrograph was obtained at an original magnification of 400× (0.29 mm^2 ^field of view). Scale bar = 50 μm.

A 23-year old woman was referred for assessment of cough, wheeze, shortness of breath, and chest tightness. She had frequent bloating, belching and loose stools. The symptoms had started two years prior to presentation with new onset sinus congestion, cough, wheeze and 40lb weight loss. Shortly after returning from a trip to Belize in the summer of 2008, her symptoms worsened and were associated with peripheral eosinophilia (4.9 × 10^9^/L) and diffuse peripheral pulmonary infiltrates. She had some features of chronic eosinophilic pneumonia; however, there was no clinical or laboratory evidence of vasculitis or hypereosinophilic syndrome (table 1). She did not have evidence of lymph node enlargement, organomegaly or skin lesions. Her FEV_1 _and VC were 1.2 L (40% predicted) and 2.4 L (65% predicted) without any further improvement with a bronchodilator. Sputum was induced with hypertonic saline and processed as described by Pizzichini et al [5] and showed 80% eosinophils. She was treated with high dose of prednisone, inhaled and nasal corticosteroids and had bilateral ethmoidectomy, sphenoidectomy and nasal polypectomy. Over the course of the next 12 months, her FEV_1 _improved to 2.1 L and her PC_20 _methacholine was 4.8 mg/ml when her sputum eosinophils were <1% on a maintenance dose of 12.5 mg daily prednisone and fluticasone+salmeterol (500+50 mcg) daily. In December 2009, she presented with severe abdominal pain, vomiting, diarrhoea and weight loss. Her blood eosinophil had risen to 3.5 and her sputum showed 28% eosinophils. FEV_1 _had declined to 1.5 L. Colonoscopy and gastroscopy revealed shallow ulcers in antrum, jejunum, caecum and rectum. Multiple biopsies were taken and Hematoxylin-Eosin (H&E) stained sections were examined independently by two pathologists who identified only a limited eosinophil infiltration of the gastrointestinal mucosa that was not consider pathological (Figure 1). However, subsequent staining of the same tissue biopsies with *EPX-mAb *[4] revealed a significant and widespread eosinophilic infiltration that was also accompanied by evidence of marked eosinophil degranulation (i.e., deposition of eosinophil peroxidase within the extracellular matrix (Figure 1). Accordingly, this patient was treated with intravenous corticosteroids with complete resolution of symptoms and improvement of FEV_1 _to 2.0 L. Subsequently, she was treated with imatinib and later with hydroxyurea, both of which failed to have any prednisone-sparing effect. She declined treatment with interferon-alpha. She is currently on 35 mg daily prednisone in addition to fluticasone+salmeterol 500+50 mcg twice daily, awaiting approval for treatment with mepolizumab, a monoclonal antibody against interleukin 5 (IL-5) [6,7].

**Table 1:** Investigations for persistent airway eosinophilia

Clinical measurement	2008	2010
Blood eosinophil (×10^9^/L)	4.9	5.3

Total serum IgE (IU/L)	1500	110

ANA, c-, p-ANCA	Not detected	Not detected

Aspergillus, farm, bird precipitins	Not detected	Not detected

Serum B12, pg/ml	300	258

Serum LDH, IU/L	124	105

Serum tryptase, IU/L	5	3

Serum TARC, pg/ml	Not done	360

Sputum IL-5, pg/ml	Not done	220

Stool for parasites (×3)	Not detected	Not detected

Toxocaris, Strongyloides serology	Negative	Negative

Sinus CT	Pan sinusitis	Pan sinusitis, polyps

Chest CT	Airspace consolidation	Minimal airspace, no nodes

Bone marrow	Normal	No clonal abnormalities

T-receptor rearrangements	Not done	Not detected

PDGFR-FIP1L1, c-kit, abl-bcr	Not done	Not detected

Cardiac MRI	Mild global hypokinesia	Normal, mitral valve prolpase

Skin biopsy	Eosinophils, no vasculitis	Not done

Colon biopsy	Normal, no vasculitis	Eosinophils, no vasculitis

Bronchoscopy	BAL eosinophils 6%	Not done

## Discussion

This clinical case provides an example of a unique association of eosinophilic gastroenteritis with eosinophilic bronchitis and asthma in the absence of atopy, vasculitis or classical hypereosinophilic syndrome. Our observations with this patient also highlight the utility of a new eosinophil-specific monoclonal antibody as a diagnostic maker of eosinophil-associated disease states.

The three clinical syndromes that may present with symptoms similar to this patient are vasculitis, chronic eosinophilic pneumonia and hypereosinophilic syndrome. Anti-neutrophil antibodies were repeatedly negative and intestinal, sinus and bronchial mucosal tissues did not show evidence of vasculitis. Although the initial radiological feature may have been consistent with chronic eosinophilic pneumonia, subsequent clinical history and radiology were not consistent with this diagnosis. Traditionally, the diagnosis is not entertained in patients who have asthma or chronic rhinosinusitis.. However, it is increasingly recognized that there is considerable overlap between the clinical and molecular patterns observed in patients with eosinophil-mediated diseases [8]. The patient did not have the classic clinical or laboratory features of myeloproliferation. Further, the mutation-related gain-of-function kinase specifically involved in the pathogenesis of myeloproliferative HES (eg, FIP1L1/PDGFRA) was not detected. However, the patient had raised levels of the eosinophilopoietic cytokine IL-5 in sputum (R&D, Mississauga, ON) and the T-cell derived eosinophilopoietin, TARC, in serum (Calbiochem, Mississauga, ON). However, we were unable to demonstrate T-cell populations in peripheral blood characterized by TCRα/β-CD3-CD4+ or CD3+CD4-CD8- that are described in patients with lymphocytic forms of HES [9]. Bone marrow examination did not show any clonal expansion of lymphocytes or eosinophils. Overall, we believe that the patient may have had a variant of a lymphocytic hypereosinophilic syndrome given the systemic eosinophilia, modestly high levels of sputum IL-5 and serum TARC and raised serum total IgE early in the course of the disease. It is possible that an unidentified allergen triggered eosinophil expansion in the bone marrow through an IgE-mediated or a non-IgE-mediated, direct T-cell interaction.

The second novel aspect of this case report is the use of a novel monoclonal antibody to identify eosinophilic infiltration of the gut. The robust character of this novel antibody (specificity and sensitivity) [4] proved invaluable to the establishment of an appropriate diagnosis by detecting both infiltrating eosinophils and the presence of eosinophil degranulation when conventional eosin and hematoxylin staining of the tissue was not interpreted as being significant by two independent pathologists. The other eosinophil granules such as ECP [10] and EDN [11] are not specific to eosinophils, being present on neutrophils. The cationic character of MBP, together with its propensity to "stick" to virtually any substratum as well as its near insolubility in environments at neutral pH limits its utility for immunohistochemistry [12]. Moreover, these intensely staining local aggregates may give the perception of eosinophil degranulation. In contrast, the nominal cationic character of EPX together with its greater solubility at neutral pH would prevent aggregation and allow this granule protein to disperse to a greater extent.

The third objective of this case report is to describe our protocol to evaluate patients with asthma who have persistent airway eosinophilia identified as sputum eosinophils >3% on two or more occasions (table 1). The investigations include workup for atopy, vasculitis, allergic bronchopulmonary aspergillosis, chronic eosinophilic pneumonia and HES. In addition, we also evaluate for hyperplastic chronic rhinosinusitis and non-IgE mediated eosinophilia possibly mediated by antigen-triggered IL-5 release from T-lymphocytes. We also recommend an assessment of steroid pharmacokinetics to monitor compliance and gastrointestinal absorption of ingested corticosteroids.

In summary, this case describes a patient who likely has a lymphocytic variant of hypereosinophilic syndrome that resulted in eosinophilic infiltration of the gastrointestinal tract, sinuses, and airway that contributed to variable airflow obstruction. The case history also illustrates the diagnostic workup of a patient with asthma who has a prednisone-dependent airway eosinophilia. The use of a novel *EPX-mAb *provided a reliable method to identify eosinophils in the gastrointestinal tract. Further research is necessary to identify the triggers for eosinophilia in L-HES and the application of the novel monoclonal antibody directed against eosinophil peroxidase to detect eosinophil activity in the airway.

## Consent

Written informed consent was obtained from the patient for publication of this case report and accompanying images. A copy of the written consent is available for review by the Editor-in-Chief of this journal.

## Competing interests

The authors declare that they have no competing interests.

## Authors' contributions

PN conceived the report and provided clinical care, JL, NL, CP and SO performed all the immunohistochemistry, ES assisted with the immunological measurements. All authors have read and approved the manuscript.
